# Leef: environmental neuroscience modeling for cognitive resilience

**DOI:** 10.3389/fnhum.2026.1756589

**Published:** 2026-04-07

**Authors:** Egzona Morina

**Affiliations:** 1 Environmental Neuroscience Research Incubator, Xheladin and Xhufe Morina Foundation, Boston, MA, United States; 2 ENRI Tech Inc., Newark, DE, United States

**Keywords:** cognitive resilience, cognitive strain, environmental neuroscience, neuro-environmental modeling, neuropredictive exposome

## Abstract

Environmental exposures, including noise, air pollution, and heat, produce measurable alterations in neural dynamics, yet these effects remain difficult to monitor outside laboratory settings. Building on recent advances in environmental neuroscience and the Neuropredictive Exposome, we introduce Leef (Local Environmental Exposure Framework), a proposed framework designed to estimate cognitive strain and adaptive capacity from real-world environmental and physiological signals. Leef integrates continuous sensing of ambient exposures (e.g., PM_2.5_, VOCs, temperature, noise) with neural signatures of environmental stress derived from controlled EEG studies, and is intended to help surface “hidden” cognitive costs that may arise even when behavioral performance appears stable. The framework formalizes how neuro-environmental relationships can be embedded into predictive modeling pipelines designed for edge-based, privacy preserving deployment. Initial implementation and prototyping are underway through ENRI Tech Inc., supporting translation of the framework into scalable research and digital health tools while remaining distinct from clinical validation at this stage. While this work does not present a validated system, it outlines the theoretical, computational, and ethical foundations required to enable real-time inference of environmentally driven cognitive strain. By linking mechanistic neuroscience to deployable sensing architectures, Leef provides a scientifically grounded pathway for studying and mitigating environmental impacts on brain function in naturalistic contexts.

## 1 Introduction

Environmental exposures such as air pollution, noise, and heat are increasingly recognized as key determinants of neurological and psychological health, with disproportionate impacts on low- and middle-income countries. While the cardiopulmonary consequences of these exposures are well documented, their moment-to-moment influence on cognitive function and affective regulation remains comparatively underexplored (Block and Calderon-Garciduenas, 2009; Bhui et al., 2023; Xiang et al., 2025; Arjunan and Rajan, 2020). Emerging findings from environmental neuroscience indicate that exposure to fine particulate matter (PM_2.5_), volatile organic compounds (VOCs), ambient noise, and thermal load can modulate large-scale neural dynamics even in the absence of overt behavioral change (Berman et al., 2019; Calderon-Garciduenas et al., 2015). This growing neuro-behavioral dissociation suggests that cognitive strain generated by environmental exposures may accumulate without obvious performance deficits, contributing to fatigue, attentional instability, and reduced wellbeing over time. A number of established frameworks have been developed to characterize environmental exposure and human health risk. The exposome paradigm conceptualizes health as shaped by the totality of environmental exposures across the lifespan (Wild, 2005). Complementary models of allostatic load describe how cumulative stressors produce long-term physiological and cognitive consequences through repeated adaptive responses (McEwen and Stellar, 1993). In the last decade, these foundational concepts have evolved into more operational and data-driven frameworks. The urban exposome and digital exposome approaches now integrate high-resolution environmental sensing, geospatial analytics, and personal health data to quantify exposure to wellbeing relationships in real-world contexts (Nieuwenhuijsen, 2020; Vermeulen et al., 2020). More recently, large-scale bibliometric analyses have demonstrated rapid growth in exposome research and increasing integration of digital data streams and multi-scale modeling (Petit and Vuillerme, 2025), while emerging work has proposed the systematic incorporation of digital behavioral traces into exposure science (Pagliaccio et al., 2024). In parallel, environmental neuroepidemiology and neuro-urbanism have begun to directly link air pollution, noise, heat, and built environments to changes in brain structure, functional connectivity, and cognitive trajectories (Yuan et al., 2023; Askari et al., 2025), providing convergent evidence that environmental stressors are reflected not only in health outcomes but in measurable neural organization itself. While these advances have substantially deepened environmental health research, most existing frameworks continue to treat the brain primarily as a downstream outcome rather than as an actively regulating, dynamically adapting system. In contrast, environmental neuroscience emphasizes that neural regulation itself is continuously shaped by environmental context, motivating the need for frameworks that explicitly integrate neural dynamics and cognitive function into exposure modeling. Yet a major translational gap remains between these scientific insights and tools capable of operating in everyday environments. Despite growing recognition of environmentally driven neural modulation, translating environmental neuroscience into real-world cognitive health applications remains challenging. Neuroimaging modalities such as EEG, fMRI, and MEG offer high temporal or spatial resolution but typically require trained personnel, controlled laboratory conditions, and substantial infrastructure. Conversely, digital health platforms that track physiological (heart-rate variability, electrodermal activity) or environmental (noise, PM_2.5_, temperature) variables often treat these channels in isolation and rarely embed neuroscience-derived targets, limiting their ability to estimate cognitive strain or adaptive capacity in context. Nonetheless, recent work has begun combining low-cost wearables with environmental monitoring to estimate stress and affect in naturalistic learning environments (Nguyen et al., 2025). In parallel, systematic reviews of the built-environment literature highlight a growing use of physiological measures and machine learning to quantify how architectural and urban contexts shape perception and wellbeing (Li et al., 2025).

To address this translational gap, we introduce the Local Environmental Exposure Framework (Leef), a developing architecture inspired by the Neuropredictive Exposome (Morina, 2025b). Leef formalizes how continuous environmental inputs can be combined with physiological markers and neuroscience-derived models to estimate cognitive strain in daily life. Recent digital exposome approaches have demonstrated the feasibility of quantifying urban environment-wellbeing relationships using sensor fusion and predictive modeling (Johnson et al., 2023). Leef builds on this trajectory by explicitly anchoring inference targets in environmental neuroscience, including oscillatory signatures of attentional regulation and cognitive strain. Empirical evidence motivates this focus. EEG-based studies have shown that noise exposure is associated with changes in spectral power features linked to attention, stress, and mental workload (Astuti et al., 2024). More broadly, controlled environmental neuroscience experiments demonstrate that adverse environmental conditions can reorganize cortical dynamics even when task performance remains stable (Berman et al., 2019; Calderon-Garciduenas et al., 2015). For example, in our recent pilot EEG study, we found that urban noise elevated θ/α power ratios relative to natural soundscapes despite similar behavioral accuracy, indicating increased cognitive effort under adverse auditory conditions (Morina, 2025a). Conversely, natural soundscapes were associated with more efficient neural states characterized by increased α and β activity, consistent with reduced cognitive strain. Importantly, noise is not the only environmental stressor shown to exert rapid neural and cognitive effects. Chemical exposures in indoor environments play a critical role: controlled exposure studies demonstrate that elevated carbon dioxide and volatile organic compound (VOC) levels are associated with significant reductions in cognitive function across executive and decision-making domains (Allen et al., 2015). Similarly, population-based evidence indicates that long-term exposure to low-level air pollutants is associated with impaired cognitive performance, particularly among older adults, with indoor air quality further modulating this relationship (Chen et al., 2023). Thermal stress likewise imposes measurable cognitive burdens. A recent meta-analysis highlights the substantial impact of heat exposure on urban health, underscoring the growing relevance of thermal stress as a public health and cognitive risk factor (Yang et al., 2024). More directly, acute heat stress has been shown to degrade attention, working memory, and decision-making performance, particularly in high-demand occupations, providing convergent evidence that extreme temperatures can impair cognitive regulation even over short timescales (Thompson et al., 2024). Together, these findings indicate that diverse environmental stressors, chemical and thermal, in addition to auditory, can impose measurable cognitive costs through partially overlapping neurophysiological mechanisms.

## 2 Conceptual framework

### 2.1 Environmental neuroscience research incubator

**Figure 1:**
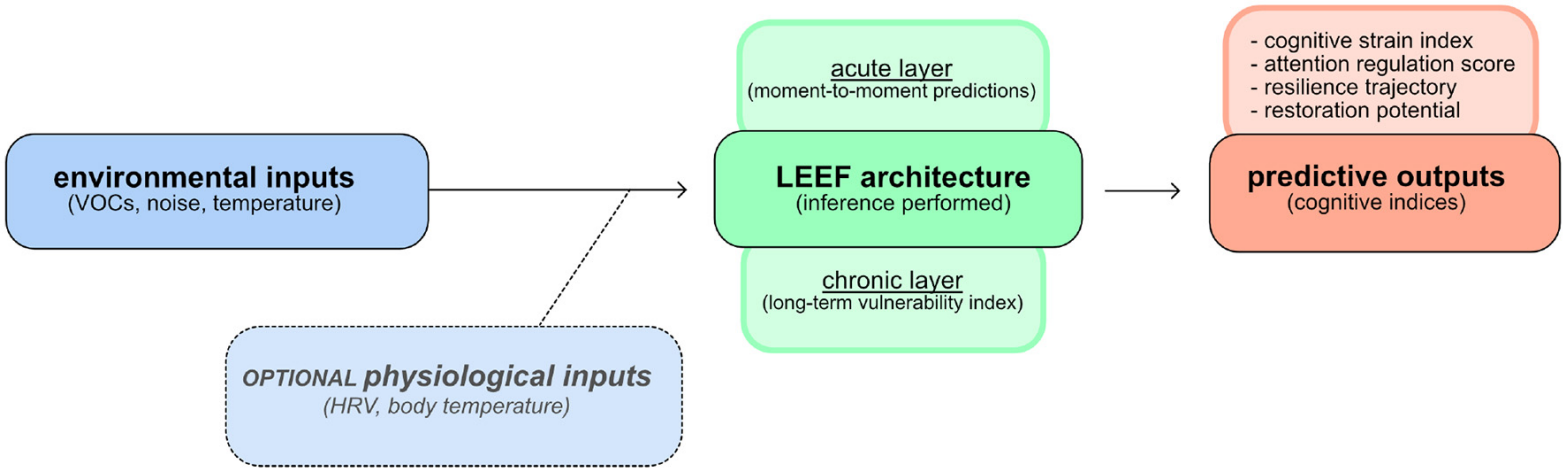
The local environmental exposure framework (Leef). The overall system integrates (1) sensor inputs (environmental and physiological data streams)(blue), (2) the Leef modeling architecture (green), comprising acute and chronic models that predict cognitive strain, and (3) a predictive outputs dashboard (orange) that presents user-facing indices and alerts. In this manuscript, Leef refers specifically to the modeling framework (green). The sensor and insight layers are depicted alongside the core modeling layer to illustrate the full end-to-end flow from environmental exposure to interpretable cognitive outputs.

The Environmental Neuroscience Research Incubator (ENRI) provides the empirical and methodological foundation for the developing Local Environmental Exposure Framework (Leef) (Figure 1). ENRI serves as a controlled experimental platform designed to systematically characterize how real-world environmental exposures shape neural regulation and cognitive control under well-defined conditions. Through a series of laboratory-based EEG studies, ENRI investigates how environmental contexts, particularly auditory environments such as urban noise and natural soundscapes, modulate large-scale cortical dynamics across frequency bands and functional networks. Rather than functioning as a deployment system, ENRI plays a critical role as a calibration and grounding layer for Leef: it generates high-fidelity neural baselines that define how specific environmental patterns map onto changes in attentional regulation, cognitive strain, and affective control. This role is central to Leef's architecture. Instead of requiring continuous neurophysiological recording in real-world settings, Leef leverages ENRI-derived neural signatures during the model development and training phase only. These signatures are used to parameterize and validate transfer functions linking environmental inputs (e.g., noise profiles, thermal load, air quality) to predicted cognitive strain and adaptive responses. During deployment, cognitive inference is performed exclusively from environmental and physiological inputs, enabling naturalistic monitoring while avoiding the invasiveness and impracticality of continuous brain recording. In this sense, ENRI functions as a bridge between controlled environmental neuroscience and scalable, real-world cognitive modeling. It allows Leef to remain empirically grounded in neural mechanisms while operating in contexts where direct brain measurement is neither feasible nor desirable. This approach reflects an emerging paradigm in environmental neuroscience: using high-resolution neurophysiological data to inform models that generalize beyond the laboratory, rather than to build systems dependent on continuous neural sensing.

### 2.2 Multimodal integration architecture

Leef is conceived as a developing modeling framework that integrates environmental sensing, physiological monitoring, and computational inference to estimate cognitive strain in naturalistic settings. While the overall system includes sensor and user-facing layers, in this manuscript “Leef” refers specifically to the modeling core that transforms environmental and physiological inputs into cognitively meaningful outputs. Conceptually, the architecture consists of three interacting layers: (1) a cognitive insights layer, (2) a sensor layer, and (3) the Leef modeling layer.

#### 2.2.1 Cognitive insights layer

The cognitive insights layer translates outputs from the acute and chronic models into interpretable indices that summarize predicted cognitive state and adaptive capacity. These indices are designed to bridge environmental measurements and neuroscience-informed interpretations of cognitive function. They are not intended as clinical or diagnostic tools, but as research-oriented constructs that can support hypothesis testing, experimental design, and user-centered exploration of environmental cognitive burden. These indices define the primary predictive targets of the Leef modeling layer, which is designed to estimate how both short-term perturbations and long-term exposures shape cognitive regulation.

#### 2.2.2 Sensor layer

The sensor layer captures continuous environmental exposures relevant to cognitive and affective regulation, including particulate matter (PM_2.5_), CO_2_, volatile organic compounds (VOCs), ambient noise levels [dB(A)], temperature, humidity, and light exposure. When available, physiological data such as heart rate variability, electrodermal activity, and sleep metrics can be integrated to provide contextual markers of autonomic regulation and recovery. These inputs are non-invasive, low-burden, and compatible with smartphone- or wearable-based deployment.

#### 2.2.3 Modeling layer

The modeling layer is formulated as a dual-pathway architecture that distinguishes acute from chronic environmental effects, consistent with principles from environmental neuroscience, allostatic load theory, and adaptive regulation frameworks. This separation reflects the well-established distinction between short-term perturbations and long-term exposure histories, which operate on different timescales and engage partially distinct neural mechanisms.

- Acute model. The acute model captures transient cognitive strain arising from rapid environmental fluctuations, such as sudden noise bursts, sharp thermal shifts, or short-term pollution elevations. These perturbations engage fast neural and autonomic responses mediated by arousal, attentional orienting, and stress-related pathways. Empirical studies demonstrate that abrupt increases in ambient noise elevate cortical θ/α power ratios, reflecting increased attentional effort even when task performance remains stable (Arjunan and Rajan, 2020; Astuti et al., 2024; Morina, 2025a). Functionally, the acute model operates on high-resolution temporal inputs and applies learned transfer functions that map momentary environmental deviations to predicted changes in cognitive strain and attentional regulation.
- Chronic model. The chronic model is designed to estimate baseline adaptive capacity under sustained environmental load by aggregating repeated exposures across longer periods, such as habitual air quality, neighborhood noise profiles, or recurrent heat stress. Rather than predicting momentary strain, it captures slowly varying shifts in cognitive resilience associated with cumulative environmental burden. Epidemiological and neuroimaging studies link long-term exposure to pollution and noise with cortical thinning, altered functional connectivity, and executive dysfunction (Bhui et al., 2023; Calderon-Garciduenas et al., 2015; Xiang et al., 2025). Algorithmically, the chronic model operates on temporally aggregated exposure metrics and incorporates dose-response relationships informed by this literature.

Although the acute and chronic models operate on distinct temporal scales, they are conceptually linked: the chronic model provides a pathway to a baseline resilience context that shapes how strongly an individual is expected to respond to acute perturbations, while the acute model is intended to capture moment-to-moment deviations from that baseline. Together, they could enable context-aware cognitive inference grounded in both instantaneous stressors and long-term adaptive capacity.

At present, these models represent a theoretically grounded framework derived from existing empirical literature and implemented in early prototype form. Although functional components of the architecture have been developed, the models are not yet presented as validated predictive tools, but rather as a structure designed to support systematic empirical evaluation and iterative refinement.

### 2.3 Predictive outputs

Leef produces a set of interpretable cognitive health indices derived from its dual-model architecture. Each index reflects a distinct aspect of cognitive regulation under environmental exposure and is normalized relative to an individual's baseline. In the Leef framework, all indices are primarily derived from environmental sensor data, optionally contextualized by passive physiological signals when available (e.g., heart rate variability, electrodermal activity). Importantly, the indices are not computed from task performance metrics or subjective questionnaires during deployment, but are inferred from environmental and physiological context alone, based on mappings learned from controlled environmental neuroscience experiments. Behavioral tasks and self-reports may be used during model training and validation, but not as required inputs for real-time inference.

- Cognitive Strain Index: the Cognitive Strain Index (CSI) is a composite estimate of the neural effort required to maintain cognitive performance under current environmental conditions. CSI reflects compensatory neural recruitment rather than task failure, allowing detection of elevated cognitive strain even when behavioral accuracy remains intact. Conceptually, CSI integrates predictions derived primarily from environmental inputs (e.g., noise levels, air quality, thermal load), optionally modulated by physiological markers of autonomic effort such as heart rate variability or electrodermal activity when available. Elevated CSI indicates that additional cognitive resources are being recruited to sustain performance, whereas lower values indicate operation within comfortable capacity. This construct aligns with evidence from environmental neuroscience showing that adverse conditions can induce energetically costly neural states without overt behavioral impairment. For example, EEG studies have demonstrated increased θ/α power ratios under noisy environments despite stable task accuracy, reflecting heightened cognitive effort (Morina, 2025a; Astuti et al., 2024). CSI is intended to capture this hidden cognitive strain and is analogous to mental workload indices used in human factors research (Wickens et al., 2021; Arico et al., 2017), while explicitly incorporating environmental context.
- Attention regulation score: the Attention Regulation Score (ARS) is designed to estimate the stability and effectiveness of attentional control under current environmental conditions. It is informed by predicted changes in neural oscillatory balance, particularly ratios involving theta, alpha, and beta power (e.g., θ/α, θ/β), which are widely used markers of attentional engagement and executive control. Lower ARS values indicate reduced attentional regulation and increased susceptibility to distraction, whereas higher values indicate stable focus and effective top-down control. Elevated theta relative to alpha or beta has been associated with mind-wandering, drowsiness, or attentional lapses, while lower ratios are characteristic of sustained engagement. These relationships are well documented in both cognitive workload and clinical attention research, including studies of attention-deficit disorders where elevated θ-to-β ratios are a hallmark of impaired regulation (Clarke et al., 2001; Arns et al., 2013).
- Resilience trajectory: the Resilience Trajectory provides a pathway to a short-term forecast of how cognitive capacity is expected to evolve over the subsequent hours given ongoing and projected environmental exposure. Rather than describing instantaneous state, this index captures the direction and momentum of cognitive regulation over time. The trajectory is derived by combining baseline adaptive capacity from the chronic model with trends in acute strain, effectively estimating how cumulative environmental demand may lead to fatigue, stabilization, or recovery. Both components are estimated from longitudinal environmental exposure profiles, optionally contextualized by passive physiological recovery signals (e.g., sleep regularity or resting HRV), without requiring repeated cognitive testing. For example, sustained exposure to noise, heat, or pollution may result in a downward trajectory indicating increasing vulnerability later in the day, whereas anticipated environmental relief may predict stabilization or recovery. This construct is motivated by evidence that environmental stressors have delayed and cumulative cognitive effects (McEwen and Stellar, 1993; Lupien et al., 2009; Bhui et al., 2023). Acute disruption can impair attention or memory hours later, and repeated exposure accelerates cognitive decline across longer timescales (Bhui et al., 2023; Xiang et al., 2025). The Resilience Trajectory thus supports proactive reasoning about cognitive sustainability rather than reactive assessment alone.
- Restoration potential: the Restoration Potential is designed to estimate the degree of cognitive benefit that could be achieved through environmental improvement, such as reduced noise, improved air quality, or exposure to natural elements. This index reflects the expected reduction in cognitive strain and improvement in attentional regulation relative to current conditions. Restoration Potential is computed by contrasting predicted cognitive state under present environmental inputs with predictions under a more restorative reference environment. This contrast is computed entirely from environmental feature space (e.g., modeled transitions from noisy to quiet environments, or from polluted to clean air), rather than from changes in observed task performance or subjective reports. Larger values indicate greater opportunity for cognitive recovery through environmental change, whereas smaller values indicate that current conditions are already near optimal. This concept is grounded in environmental psychology and neuroscience research demonstrating that restorative environments, particularly natural or low-stimulation settings, reduce mental fatigue and improve cognitive performance. Attention Restoration Theory proposes that exposure to nature replenishes depleted attentional resources, a claim supported by experimental findings showing improved memory, attention, and neural efficiency following interaction with natural environments (Kaplan, 1995; Berman et al., 2008). Environmental neuroscience studies similarly show that natural soundscapes promote more efficient cortical dynamics compared to urban noise (Berman et al., 2019; Morina, 2025a). Restoration Potential formalizes these effects into a quantitative, individualized estimate.

All cognitive indices are designed to be interpretable, non-diagnostic, and privacy-preserving. Inference relies primarily on environmental sensor data, with optional incorporation of passive physiological signals, and does not require continuous cognitive testing or subjective questionnaires during deployment. Data processing occurs locally on edge-computing platforms, ensuring that environmental and physiological signals remain under user control and are not transmitted externally without explicit consent.

## 3 Applications and societal impact

### 3.1 Cognitive health

Potential applications span multiple settings. In workplaces, Leef could assist users in identifying lower-strain environments within buildings (e.g., quieter zones or areas with improved air quality) and in planning work schedules to avoid periods of high pollution or disruptive noise, consistent with evidence that indoor environmental quality directly shapes cognitive performance and fatigue (Allen et al., 2015; MacNaughton et al., 2017). In educational contexts, the framework may support more effective learning environments by highlighting exposure conditions associated with improved attentional stability, aligning with growing evidence that noise, air quality, and thermal comfort influence learning outcomes and executive functioning (Shield et al., 2010).

As environmental change intensifies the frequency and severity of heat events, estimating the cognitive impact of elevated temperature becomes increasingly relevant for public health, particularly for groups with heightened vulnerability such as outdoor workers, older adults, and individuals with limited access to cooling resources (Obradovich et al., 2017). Heat exposure has been linked not only to reduced labor productivity but also to impaired decision-making and increased error rates, underscoring the importance of integrating cognitive metrics into climate adaptation strategies (Graff Zivin et al., 2018).

A central principle of the Leef framework is individualized modeling. Rather than relying on neurotypical or population-level baselines, Leef adapts its estimations to each user's exposure history and physiological profile, supporting a more inclusive approach to cognitive health monitoring. This design accommodates variability in sensory sensitivity, environmental preferences, and adaptive capacity, consistent with emerging personalized environmental health paradigms. Initial pilot deployments in Boston/Cambridge (USA) and Prishtina (Kosovo) will evaluate the feasibility of implementing the framework across distinct socioeconomic and environmental contexts. These pilots will inform its further development as a scalable tool for supporting cognitive sustainability in diverse communities.

### 3.2 Data democratization and global equity

A central motivation behind developing Leef is to extend the reach of environmental neuroscience beyond laboratory settings and high-resource institutions. By relying on accessible sensors rather than specialized neuroimaging equipment, Leef supports the democratization of cognitive health insights. The framework is intentionally designed to operate on widely available, low-cost platforms, such as smartphone-integrated sensors and affordable air quality or noise monitors, making it adaptable to regions where traditional neuroimaging infrastructure is limited or absent (Nieuwenhuijsen, 2020).

This scalability is particularly important for low- and middle-income countries, where environmental exposures such as air pollution, noise, and heat are often higher, yet fewer tools exist to quantify their cognitive consequences. Environmental justice research has consistently shown that marginalized communities bear a disproportionate burden of environmental stressors, including higher exposure to air pollution and urban heat islands (Hsu et al., 2021; Mikati et al., 2018). These disparities are increasingly linked not only to physical health outcomes but also to cognitive development, mental health, and educational attainment (Calderon-Garciduenas et al., 2015).

By enabling individuals, workplaces, and public health agencies to monitor environmental conditions that influence cognitive wellbeing, Leef promotes a more equitable distribution of scientific and technological benefits. The long-term goal is to support evidence-based decision-making in communities that face disproportionate environmental burdens, thereby contributing to global efforts toward cognitive and environmental justice (Brulle and Pellow, 2006). In this way, Leef is positioned not only as a technological framework, but as an enabling infrastructure for more just and cognitively sustainable urban and occupational environments.

## 4 Ethical and design principles

### 4.1 Privacy-preserving architecture

A central design requirement for the developing Leef architecture is strict protection of participant data. To minimize privacy risks associated with continuous environmental and physiological sensing, all preliminary prototypes are implemented using local processing on edge-capable devices. No raw data are transmitted off-device by default, aligning with recommendations for privacy-preserving digital health tools. Any optional data sharing for research validation requires explicit, study-specific consent, and all shared data undergo de-identification procedures consistent with current ethical guidelines.

### 4.2 Human-centered design and informed consent

Given that environmental sensing is designed to reveal sensitive patterns about daily life, the framework is being developed with an emphasis on user autonomy and transparency. Participants are informed about what data streams are collected, how environmental and physiological variables are interpreted within the modeling framework, and the limitations of the system's predictive capacity. Importantly, all cognitive-health indicators generated by this research architecture are intended as informational tools rather than diagnostic outputs. This distinction is communicated clearly during consent to ensure that participants do not interpret predictions as clinical evaluations.

### 4.3 Minimizing algorithmic and environmental cost

The computational design of Leef prioritizes efficiency to reduce both model bias and computational carbon load. Unlike large black-box neural networks, the architecture focuses on lightweight models trained using domain-constrained predictors informed by environmental neuroscience. This reduces the energy requirements for training and inference while improving interpretability. Model compression and on-device inference techniques are explored to further minimize computational overhead, which is particularly important for field-based research deployments in resource-constrained settings.

### 4.4 Open science and transparent validation

Although certain components of the architecture require protection during development (e.g., for patent evaluation), the scientific foundation is grounded in open research principles. Environmental neuroscience findings motivating the architecture, along with validation studies, are intended to be published in peer-reviewed venues. Where possible, de-identified validation datasets and analysis code will be shared to facilitate reproducibility, consistent with emerging standards in computational neuroscience. This approach allows the broader scientific community to scrutinize, replicate, and refine the modeling assumptions underlying Leef, ensuring that its development aligns with rigorous scientific and ethical norms.

### 4.5 Limitations and next steps

Leef is currently a theoretical and early-prototype framework rather than a validated predictive system. The manuscript does not report model performance metrics, does not validate indices at the individual level against concurrent neural or behavioral ground truth in real-world settings, and does not establish clinical utility. The mapping from environmental features to inferred cognitive states is therefore provisional and must be tested empirically. The necessary next step is to collect longitudinal, real-world individual data (environmental + optional physiology, with structured validation tasks and/or EEG ground truth in controlled subsets) to calibrate model parameters, assess generalizability across populations and contexts, quantify uncertainty, and evaluate whether Leef's indices track meaningful within-person variation over time.

## 5 Conclusion and outlook

Leef proposes a developing architecture that translates emerging insights from environmental neuroscience into an accessible framework for estimating cognitive strain and adaptive capacity using real-world environmental and physiological inputs. By integrating environmental sensing with predictive modeling, Leef offers a pathway toward continuous, context-aware monitoring of cognitive health without the need for direct neural recording during deployment. The system's dual-model structure provides a pathway to a mechanistic basis for understanding how different environmental conditions impose cognitive strain. Its privacy-preserving, edge-based processing strategy further supports real-world feasibility by keeping sensitive data under user control while enabling meaningful interpretation of the interaction between the environment and cognition. Looking ahead, Leef has potential applications across scales: from individual self-regulation and workplace design to urban planning and public health initiatives. The framework can inform interventions aimed at reducing cognitive strain, improving attentional stability, and enhancing daily performance. Validation studies across culturally and environmentally distinct settings will be critical for determining generalizability, usability, and social impact. Future work should focus on validating predictive models through longitudinal and real-world studies, and developing actionable intervention protocols grounded in mechanistic insights from environmental neuroscience. There is also an opportunity to explore clinical applications, including monitoring cognitive vulnerability in neurological or psychiatric conditions and evaluating environmental modifications as therapeutic adjuncts. As environmental pressures intensify, understanding cognition in context becomes increasingly essential. Leef offers an emerging pathway toward operationalizing this principle, supporting evidence-based cognitive health management in everyday environments. The present framework does not evaluate model performance, does not claim clinical accuracy, and does not provide behavioral or neural validation, these steps are reserved for future empirical work.

## Data Availability

The data analyzed in this study is subject to the following licenses/restrictions. The dataset underlying this manuscript cannot be made publicly available because it forms part of ongoing development toward a proprietary digital health product (Leef). The data are therefore restricted for commercial and intellectual property protection. Access may be granted upon reasonable request, subject to data-use agreements and IRB approval. Requests to access these datasets should be directed to egzona@xhmf.org.
